# Feasibility of a novel self‐assembling submucosal injection peptide solution for endoscopic mucosal resection of colorectal lesions: A multicenter study

**DOI:** 10.1002/deo2.70069

**Published:** 2025-02-13

**Authors:** Keigo Sato, Takehide Fukuchi, Shinpei Kondo, Yuya Nakano, Yoko Hachisu, Kengo Kasuga, Ayako Matsui, Hironori Aoki, Kohei Takizawa, Shiko Kuribayashi, Yoji Takeuchi, Toshio Uraoka

**Affiliations:** ^1^ Department of Gastroenterology and Hepatology Gunma University Graduate School of Medicine Gunma Japan; ^2^ Department of Gastroenterology Fujisawa City Hospital Kanagawa Japan; ^3^ Department of Gastroenterology Gunma Saiseikai Maebashi Hospital Gunma Japan; ^4^ Department of Gastroenterology Isesaki Municipal Hospital Gunma Japan; ^5^ Endoscopy Center Koyukai Shin‐Sapporo Hospital Hokkaido Japan

**Keywords:** colon, colonoscopy, colorectal polyps, endoscopic mucosal resection, endoscopic resection

## Abstract

**Objectives:**

Although a novel submucosal injection material consisting of a fully synthetic, self‐assembling peptide solution, PuraLift, has recently become commercially available in Japan, there are a few reports regarding the usefulness of this solution. The aim of this study was to investigate the feasibility of PuraLift for conventional endoscopic mucosal resection (EMR) in clinical practice.

**Methods:**

This multicenter retrospective study was conducted at the endoscopy units of five institutions from January 2023 to May 2023. Consecutive patients who underwent EMR with PuraLift for 5–20‐mm colorectal lesions were included in the introduction of this solution at each institute. The primary endpoint was the “effective resection” rate, defined as pathological complete resection, with “effective injection” defined as requiring no more than one additional injection due to adequate maintenance of mucosal lifting during EMR.

**Results:**

In total, 110 lesions in 70 patients were treated by conventional EMR using PuraLift. En‐bloc resection was performed for 109 (99%) lesions, and complete resection was performed for 102 (93%) lesions. More than 95% of the lesions were neoplastic. Additional injections were required in only two lesions. Both were single additional injections, and the median overall injection volume was 1.5 mL. Therefore, the effective injection rate was 93% (95% confidence interval, 86%–96%). No adverse events occurred during the study period.

**Conclusions:**

Although direct comparison with other materials is required, PuraLift seems feasible as an injection material for EMR.

## INTRODUCTION

Endoscopic resection is one of the least invasive treatment options for precancerous lesions or early‐stage cancer without a risk of lymph node metastasis. However, residual polyps after incomplete endoscopic resection can lead to post‐colonoscopy colorectal cancer. Therefore, endoscopists have made various efforts to achieve complete local resection. Recently, cold snare polypectomy, a polypectomy procedure without electrocautery, has been performed for adenomas smaller than 10 mm. However, for lesions larger than 10 mm or those suspected to be malignant, endoscopic mucosal resection (EMR) is commonly performed.

EMR involves the injection of fluid into the submucosal layer to elevate the lesion into a polyp‐like shape, followed by snaring and resection with an electrosurgical snare. This technique improves the complete resection rate of colorectal lesions smaller than 20 mm. The submucosal injection also reduces the risk of perforation, a serious adverse event in endoscopic resection, by increasing the distance between the muscle layer and the mucosa. Several agents have been used as local injection solutions, and the search for the most effective solution continues.^1^, ^2^

PuraLift (3‐D Matrix; Tokyo), a novel submucosal injection material consisting of a fully synthetic and self‐assembling peptide solution, was recently approved under the Japanese public insurance reimbursement system. Animal studies have shown its usefulness as a submucosal injection material for EMR.^3^ We subsequently published a case report showing that PuraLift provided sufficient protrusion formation for safe endoscopic submucosal dissection.^4^ The present multicenter retrospective study was performed to investigate the feasibility of PuraLift as an injection material for conventional EMR in clinical practice.

## METHODS

### Study design and patients

This retrospective multicenter observational study was conducted at the endoscopy units of five centers (Gunma University Graduate School of Medicine, Fujisawa City Hospital, Gunma Saiseikai Maebashi Hospital, Isesaki Municipal Hospital, and Koyukai Shin‐Sapporo Hospital) from January 2023 to May 2023, shortly after approval of PuraLift by the Japanese public insurance reimbursement system on December 1, 2022. The data of consecutive patients who underwent EMR after injection of PuraLift for colorectal lesions between the introduction of this solution at each institute and May 31 were collected and analyzed. During the study period, endoscopists were recommended to use PuraLift for 5‐ to 10‐mm non‐pedunculated lesions suspected to be high‐grade dysplasia or cancer, pedunculated lesions, or 10‐ to 20‐mm lesions not suitable for cold snare polypectomy. Patients with recurrent lesions, ulcers in the lesions, inflammatory bowel disease, familial adenomatous polyposis, undergoing chemotherapy, and deemed unsuitable for use of PuraLift by the treating endoscopists at each institution underwent conventional EMR using 0.9% saline, glycerol, hyaluronate sodium,^5^ or endoscopic submucosal dissection.

The study protocol was approved by the Gunma University Hospital Clinical Research Review Board. All patients provided written informed consent to undergo EMR. Informed consent for participating in this study was obtained in the form of an opt‐out method on the website. The reporting of this study followed the Strengthening the Reporting of Observational Studies in Epidemiology Statement.^6^

### Injection material

The injection material used in this study, PuraLift, is an aqueous peptide solution in a vial, primarily composed of self‐assembling peptides at physiologic pH. When placed under physiological conditions, the peptide solution quickly forms a hydrogel comprising a network of nanofibers through contact with body fluids such as digestive and tissue fluids secreted from the stomach and intestines. The injected hydrogel remains in the submucosa, creating a wide separation between the mucosal and muscular layers. This results in the elevation of the lesion, which is maintained during EMR or endoscopic submucosal dissection.^3^

### Procedures

The endoscopic diagnoses of the lesions were based on their macroscopic appearance (Paris classification^7^), magnifying narrow‐band imaging findings (Japan Narrow‐band Imaging Expert Team [JNET] classification^8^), or pit pattern classification.^9^, ^10^ EMR was indicated for lesions with a JNET 2A/2B classification or a pit pattern other than V_N_, typically adenoma, sessile serrated lesion, and high‐grade dysplasia/intramucosal adenocarcinoma for 10‐ to 20‐mm lesions.

**FIGURE 1:**
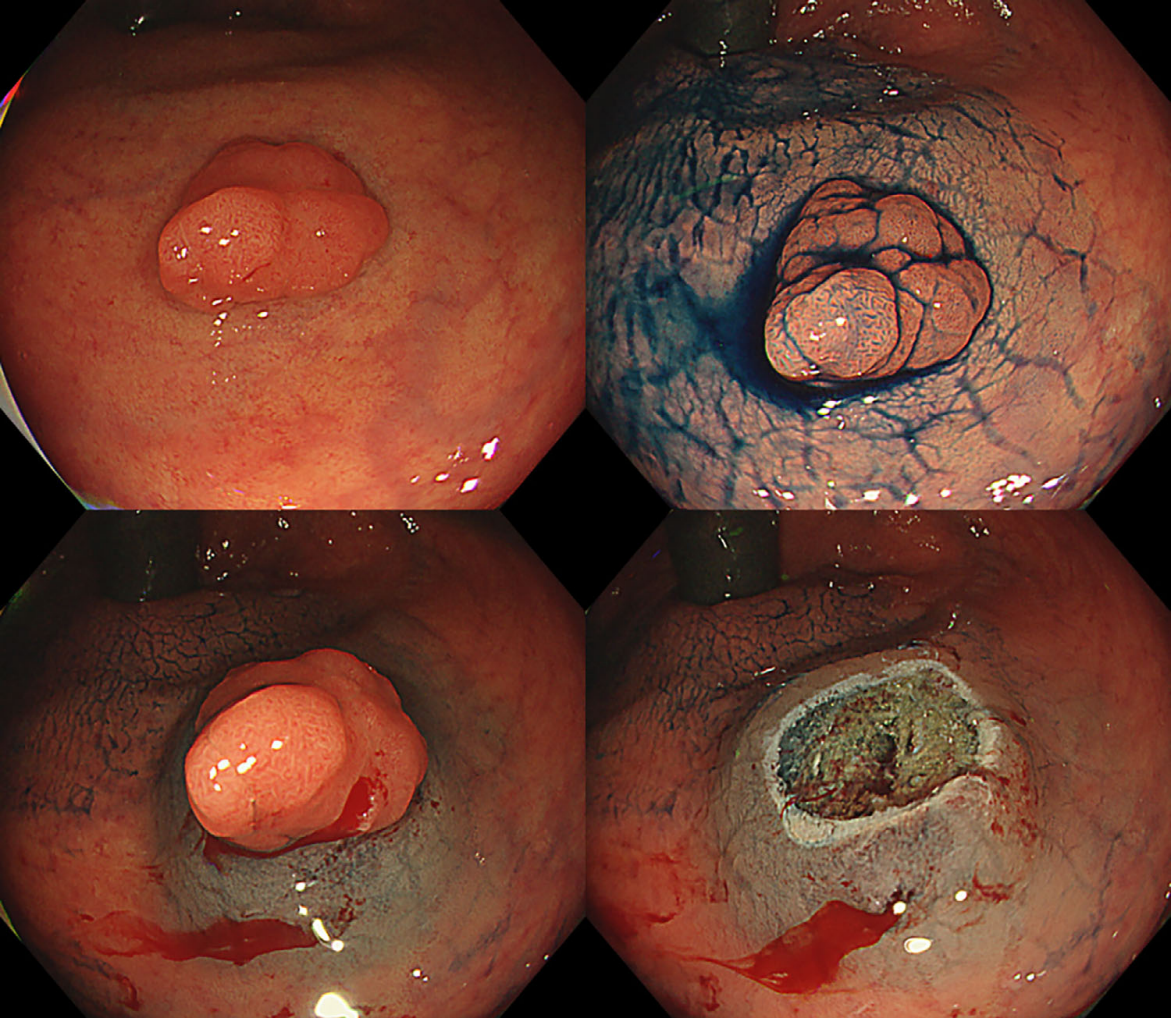
Lesion underwent endoscopic mucosal resection after injection of PuraLift.

EMR was performed according to each institution's protocol, primarily using carbon dioxide inflation. The use of a cap was left to the endoscopists’ discretion. After careful lesion assessment, PuraLift was injected to achieve adequate protrusion for EMR, allowing the lesion along with the surrounding non‐neoplastic mucosa to be entrapped by an electrosurgical snare. Adequate protrusion is generally considered as a bulge that is taller than 1 cm, has a steep rise, and has a vertical rise that is longer than its horizontal extent (Figure 1). All endoscopists attempted R0 resection during the initial resection. Multiple needle punctures without insertion and withdrawal of the endoscopic needle through the endoscope working channel were performed in one session. Additional injections were permitted and counted if the appropriate protrusion was not maintained or had shrunk after the initial injection session. After the submucosal injection of PuraLift, the mucosal protrusion, including the lesion, was entrapped with an electrosurgical snare. Electrocautery was then applied to remove the lesion. If residual lesions were observed or suspected, additional injection and resection using the same strategy were allowed until complete removal was achieved. The electrosurgical snares used in this study were chosen based on their availability in each institution. Although the electrocautery settings differ due to the different equipment and electrosurgical unit used at each facility, the main facility, Gunma University, used the end‐cut mode Effect2 40W (VIO3; Erbe Elektromedizin GmbH) for EMR, and the other facilities had similar settings.

The resected specimens were retrieved, fixed in 10% formalin solution, and embedded in paraffin. The fixed specimens were sectioned serially at 2‐mm intervals. Histological diagnoses and assessments of resection margins were made according to the Japanese Classification of Colorectal Carcinoma.^11^

### Data collection

We collected the following data from the patients’ medical records and compiled it at Gunma University Graduate School of Medicine: age, sex, lesion location, macroscopic classification,^7^ lesion size, colonoscopy experience, pathological diagnosis including evaluation of lesion involvement on the margins, number of additional injections, necessity for other injection materials, and intraoperative and postoperative adverse events. Postoperative adverse events were generally reported at an outpatient visit two weeks after treatment to explain the pathology results and any adverse events were interviewed and recorded on the medical chart.

### Endpoints and research data

The primary endpoint was the “effective resection” rate, defined as pathological complete resection, with “effective injection” defined as requiring no more than one additional injection due to adequate maintenance of mucosal lifting during EMR. Additional injection was defined as any injections performed after the initial injection to achieve sufficient elevation. The secondary outcome measures were the en bloc resection rate, number of additional injections, use of other injection material, and adverse events.

Because PuraLift was used for lesions in which cold snare polypectomy was not indicated, some of the enrolled patients had pedunculated lesions. For most pedunculated lesions, however, en bloc or complete resection was not difficult to achieve regardless of submucosal injection, and we analyzed outcomes only for non‐pedunculated lesions in a subgroup analysis.

### Sample size calculation

A performance goal was established based on a previous clinical study^12^ involving a 0.13% hyaluronic acid solution, a similar high‐viscosity injection material for colorectal EMR. In that study, the usefulness rate for assisted endoscopic resection was 81.6% (31/38), with a 95% confidence interval (CI) ranging from 65.7% to 92.3%. To estimate this usefulness rate with higher precision, the lower limit of the 95% CI was set as the goal to exceed 70%, and the number of cases needed to achieve this goal was calculated to be 62. This was a retrospective observational study, and as many cases as possible were accumulated during the study period, exceeding the calculated sample size.

### Statistical analysis

Categorical variables are presented as point estimates with 95% CIs. Quantitative data with a normal distribution are presented as mean (standard deviation). Non‐parametric data are presented as median (range).

## RESULTS

### Patient and lesion characteristics

**FIGURE 2:**
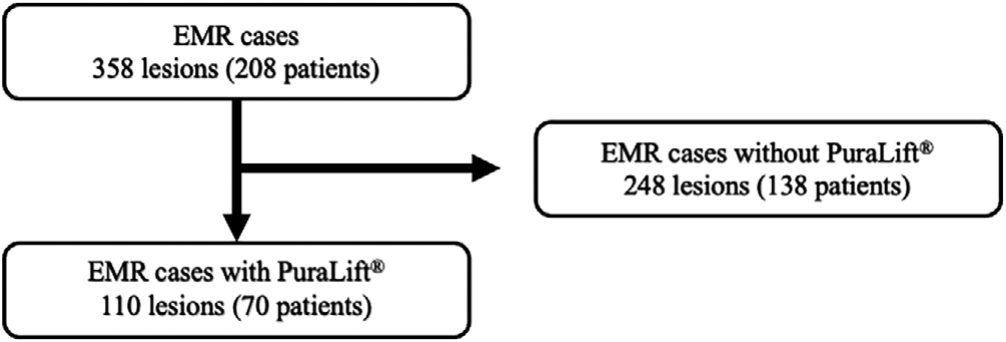
Study flowchart of the enrolled lesions.

During the study period, a total of 358 lesions in 208 patients were treated by conventional EMR. After excluding lesions and patients where injection solutions other than PuraLift were used, 110 lesions in 70 patients were treated by conventional EMR using PuraLift (Figure 2). All data were collected and analyzed at Gunma University. The characteristics of the patients and lesions are shown in Table 1. The patients’ median age was 67 years, and 61% were male. The lesions were most frequently located in the left‐sided colon, and protruded lesions were more common than superficial lesions (72% vs. 26%, respectively). Approximately one‐third of the lesions were >11 mm, and 36% of the lesions were resected by less experienced endoscopists (<1000 colonoscopy procedures).

**TABLE 1:** Patient and lesion characteristics.

Patients	*n* = 70
Age, years	67 (38–86)
Sex	
Male	43 (61.4)

Lesions	*n* = 110
Location	
Right colon	44 (40.0)
Left colon	51 (46.4)
Rectum	15 (13.6)
Morphology	
0‐Ip	19 (17.3)
0‐Is	62 (56.4)
0‐IIa	29 (26.4)
Size	
5–10 mm	73 (63.4)
11–15 mm	30 (27.3)
16–20 mm	7 (6.4)
Experience, number of procedures	
<1000	40 (36.4)
1000–2000	22 (20.0)
2001–5000	23 (20.9)
>5000	25 (22.7)

Data are presented as *n* (%) or median (range).

### Measured outcomes

Table 2 shows the treatment outcomes in this study. En‐bloc resection was performed for 109 (99%) lesions, and complete resection was performed for 102 (93%) lesions. More than 95% of the lesions were neoplastic. Additional injections were required for only two lesions. Both were single additional injections, and the median overall injection volume was 1.5 mL. No additional injection fluid was used other than PuraLift. Therefore, the effective resection rate was 93% (95% CI, 86–96). There were no adverse events related to this agent or the EMR procedure.

**TABLE 2:** Treatment outcomes.

Lesions	*n* = 110 [95% confidence interval] (%)
Number of additional injections	
0	108 (98.1)
1	2 (1.8)
Total injection amount, mL	1.5 (0.2–14)
Other injection material	
No	110 (100)
En bloc resection	
Yes	109 (99.1 [94.5–100])
Complete resection	
Yes	102 (92.7 [86.1–96.5])
Effective resection	
Yes	102 (92.7 [86.1–96.5])
Pathological diagnosis	
Adenoma	93 (84.6)
Adenocarcinoma	10 (9.1)
Intramucosal adenocarcinoma	8 (7.2)
Submucosal adenocarcinoma	2 (1.8)
Hyperplasia	3 (2.7)
Sessile serrated lesion	3 (2.7)
Inflammatory fibroid polyp	1 (0.9)
Adverse events	0 (0 [0–4.1])

Data are presented as *n* (%) or median (range).

### Subgroup analysis for non‐pedunculated lesions

The characteristics of the patients and lesions, excluding pedunculated lesions, are shown in Table 3. Age, sex, and lesion location tended to be similar to those of the overall participants. Almost three‐fourths of the lesions were 5–10‐mm in size. One‐fourth of the lesions were resected by less experienced endoscopists (<1000 colonoscopy procedures).

**TABLE 3:** Patient and non‐pedunculated lesion characteristics.

Patients	*n* = 91
Age, years	68 (38–86)
Sex	
Male	35 (61.4)

Lesions	*n* = 91
Location	
Right colon	40 (44.0)
Left colon	38 (41.8)
Rectum	13 (14.3)
Morphology	
0‐Is	62 (68.1)
0‐IIa	29 (31.9)
Size	
5–10 mm	66 (72.5)
11–15 mm	19 (20.9)
16–20 mm	6 (6.6)
Experience, number of procedures	
<1000	26 (28.6)
1000–2000	21 (23.1)
2001–5000	20 (22.0)
>5000	24 (26.4)

Data are presented as *n* (%) or median (range).

Table 4 shows the treatment outcomes excluding pedunculated lesions. The en bloc resection rate, complete resection rate, pathological diagnosis, additional injections, median injection volume, effective resection rate, and adverse events were similar to the overall results. The effective resection rate was 91% (95% CI, 83–96) for non‐pedunculated lesions.

**TABLE 4:** Treatment outcomes for non‐pedunculated lesions.

Lesions	*n* = 91 [95% confidence interval] (%)
Number of additional injections	
0	89 (97.8)
1	2 (2.2)
Total injection amount, mL	1.5 (0.2–14)
Other injection material	
No	91 (100)
En bloc resection	
Yes	90 (99.0 [93.4–100])
Complete resection	
Yes	83 (91.2 [83.4–95.7])
Effective resection	
Yes	83 (91.2 [83.4–95.7])
Pathological diagnosis	
Adenoma	79 (86.8)
Adenocarcinoma	5 (5.5)
Intramucosal adenocarcinoma	3 (3.2)
Submucosal adenocarcinoma	2 (2.2)
Hyperplasia	3 (2.73)
Sessile serrated lesion	3 (2.73)
Inflammatory fibroid polyp	1 (0.91)
Adverse events	0 (0)

Data are presented as *n* (%) or median (range).

### Subgroup analysis according to non‐pedunculated lesion size

Table 5 shows the results according to non‐pedunculated lesion size. EMR for 11–20‐mm non‐pedunculated lesions was mainly performed by experienced endoscopists (≥1000 colonoscopy procedures). En‐bloc resection and complete resection of 5–10‐mm lesions were performed in 66 of 66 (100%) cases and 62 of 66 (93.9%) cases, respectively. En‐bloc resection and complete resection of 11–20‐mm lesions were performed in 24 of 25 (96%) and 21 of 25 (84%) cases, respectively. Adenocarcinoma was seen only in 11–20‐mm lesions. Two additional counts of injection were performed in 11–20‐mm lesions. The median total injection amount was 1 mL for 5–10‐mm lesions and 3 mL for 11–20‐mm lesions. The effective resection rate was 94% (95% CI, 85–98) for 5–10‐mm lesions and 84% (95% CI, 65–94) for 11–20‐mm lesions.

**TABLE 5:** Patient and non‐pedunculated lesion characteristics and treatment outcomes according to lesion size.

Lesions	5–10 mm (*n* = 66) [95% confidence interval] (%)	11–20 mm (*n* = 25) [95% confidence interval] (%)
Location		
Right colon	29 (43.9)	11 (44.0)
Left colon	29 (43.9)	9 (36.0)
Rectum	8 (12.0)	5 (20.0)
Morphology		
0‐Is	46 (70.0)	16 (64.0)
0‐IIa	20 (30.3)	9 (36.0)
Experience, number of procedures		
≤1000	23 (34.8)	3 (12.0)
1000–2000	16 (24.2)	5 (20.0)
2001–5000	15 (22.7)	5 (20.0)
≥5000	12 (18.1)	12 (48.0)
Number of additional injections		
0	66 (100)	23 (92.0)
1	0 (0.0)	2 (8.0)
Total injection amount, mL	1 (0.2–4)	3 (1–14)
En bloc resection		
Yes	66 (100 [93.4–100])	24 (96.0 [78.9–100])
Complete resection		
Yes	62 (93.9 [85.0–98.0])	21 (84.0 [64.7–94.2])
Effective resection		
Yes	62 (93.9 [85.0–98.0])	21 (84.0 [64.7–94.2])

Data are presented as *n* (%) or median (range).

## DISCUSSION

This is the first multicenter retrospective study to evaluate the feasibility of the novel submucosal injection material PuraLift in EMR. The results indicated that PuraLift is able to feasibly assist colorectal EMR.

The primary endpoint of this study was assessed by comprehensively evaluating en bloc complete resection with the lifting and maintenance of a mucosal lesion during endoscopic resection. A similar study using hyaluronic acid solution revealed a complete resection rate of 82.5% (33/40) and an en bloc resection rate of 92.5% (37/40).^12^ Other studies of EMR using the hyaluronic acid solution for lesions other than pedunculated lesions showed complete resection rates of 78.3%–79.5% and en bloc resection rates of 93.3%–96.7%.^13^, ^14^ Although there were some minor differences compared with the present study, our results were considered generally favorable. In a similar study using a hyaluronic acid solution, additional injections were needed in only one patient, who required two additional injections.^12^ The results were similar to the present study, indicating that the bulge was generally maintained. There were no adverse events after EMR, such as bleeding or perforation. The data from this study suggest that this novel injection solution is useful in EMR because the complete resection and en bloc resection results were favorable, frequent additional injections were not required, and no adverse events associated with the solution occurred. Our data may contribute to the more widespread use of PuraLift by demonstrating its performance in a practical setting with endoscopists of varying levels of experience at community hospitals.

In the treatment of 11–20‐mm lesions using a hyaluronic acid solution, a previous study revealed complete resection and en bloc resection rates of 72.2% and 85.1%, respectively.^13^ Underwater EMR showed a complete resection rate of 69.0% and en bloc resection rates of 84.6%–89.0%.^15^, ^16^ Although these data are not directly comparable because of the small number of patients in this study, the outcomes using PuraLift appear to be at least non‐inferior to other modalities. EMR using PuraLift seems to be a good option for moderate lesions of 11 to 20 mm. Given the cost of PuraLift, selective use of PuraLift in EMR procedures is necessary. As a next step in our research, we are planning a study to further investigate the efficacy of PuraLift specifically in larger lesions, particularly those suspected to be malignant, where en bloc resection is desired.

The main strength of this study is the participation of endoscopists with different levels of experience from community hospitals, indicating the generalizability of PuraLift. However, this study also had several limitations. First, this was a retrospective study, which may have introduced selection bias. A prospective study should be conducted to validate our results. Second, this study is a single‐arm study and does not include a comparison with other injection materials. A randomized controlled trial should be conducted. Third, the procedure time was not investigated because of the retrospective study design. Underwater EMR reportedly has a shorter procedure time than conventional EMR.^17^ A shorter procedure time would naturally reduce patient discomfort; therefore, further investigation is desirable. We have several expectations regarding the use of this solution. PuraLift is a formulation similar in composition to PuraStat, which is a hemostatic agent used in endoscopic therapy. In addition, there are reports that PuraStat seems to promote ulcer healing.^18^ We expect PuraLift to have similar effects. Further investigation is warranted to determine whether PuraLift can prevent perioperative bleeding and delayed bleeding and promote the healing of ulcers after endoscopic resection. Such effects may provide advantages over other injection solutions.

## CONCLUSION

Although direct comparison with other materials is required, PuraLift seems feasible as an injection material for EMR.

## CONFLICT OF INTEREST STATEMENT

Dr. Uraoka has received consulting and lecture fees from 3‐D Matrix Ltd. PuraLift was provided by 3‐D Matrix. Dr. Uraoka serves as Deputy Editor‐in‐Chief of *DEN Open*. Dr. Takeuchi serves as Associate Editor of *DEN Open*.

## ETHICS STATEMENT

Approval of the research protocol by an Institutional Reviewer Board: The study protocol was approved by the Gunma University Hospital Clinical Research Review Board (No. 2023–033).

## PATIENT CONSENT STATEMENT

The requirement for informed consent from each patient was waived in this study.

## CLINICAL TRIAL REGISTRATION

N/A.

## Data Availability

The datasets generated and/or analyzed during the current study are available from the corresponding author upon reasonable request.
